# Influence of Implant Adjacent Teeth on the Accuracy of Digital Impression

**DOI:** 10.1055/s-0043-1771031

**Published:** 2023-08-29

**Authors:** Jaafar Abduo, Hossam El-Haddad

**Affiliations:** 1 Department of Prosthodontics, Melbourne Dental School, Melbourne University, Melbourne, Victoria, Australia

**Keywords:** edentulous area, intraoral scanner, precision, trueness

## Abstract

**Objective** The aim of this study was to evaluate the effect of adjacent teeth patterns on the accuracy of digital scans of parallel and divergent implants for three-unit prostheses.

**Materials and Methods** A maxillary typodont model with implants in the locations of the first premolars and first molars was used to develop three clinical scenarios for three-unit prostheses: (S1) Partially edentulous arch with missing first premolars and first molars only; (S2) partially edentulous arch with missing first premolars, second premolars and first molars; and (S3) partially edentulous arch with missing canines, first premolars, second premolars, first molars, and second molars. On one side, the implants were parallel, and for the other side, the implants had a 15-degree buccolingual angle. With the aid of scan bodies, 10 digital impressions were taken for each scenario and for each side. To evaluate the accuracy, a reverse engineering software was used to measure trueness, precision, and interimplant distance.

**Results** The best trueness for parallel implants was observed for S2 (30.0 µm), followed by S3 (67.3 µm) and S1 (74.8 µm) ( *p* < 0.001). Likewise, S2 had the best precision for parallel implants (31.3 µm) followed by S3 (38.0 µm) and S1 (70.3 µm) ( *p* < 0.001). For the divergent implants, S2 exhibited the best trueness (23.1 µm), followed by S3 (48.2 µm) and S1 (59.4 µm) ( *p* = 0.007). Similarly, the S2 had the best precision (12.3 µm) followed by S3 (62.1 µm) and S1 (66.9 µm) ( *p* < 0.001). The S2 had the least interimplant distance deviation followed by S1 and S3. The difference was significant for parallel implants ( *p* = 0.03), but insignificant for divergent implants ( *p* = 0.15).

**Conclusion** Regardless of the presenting scenario, digital implant impressions for three-unit prostheses appear to be clinically accurate. A clear interimplant area between scan bodies enhanced the accuracy of digital impressions. This observation can be attributed to more accessible axial surface scanning of the scan body.

## Introduction

Digital impression by intraoral scanner (IOS) is rapidly growing to become a popular method to record the position of oral implants. It has the advantages of the simplicity of the procedure, quicker recording, patient comfort, and no material wastage. In addition, digital impressions can easily be transferred virtually to the manufacturing technician, which reduces the transportation burden and the length of treatment. The recent studies indicate that the accuracy of digital implant impression is at least as accurate as conventional implant impression. 1 2 3 4 5 6 7 8 9 On the other hand, the accuracy of digital impressions of multiple implants for multiunit prostheses is more critical than for digital impressions of single implants because the prosthesis has to fit accurately and simultaneously on all the implants. 10 The lack of accurate fit between the prosthesis and the underlying implants can lead to numerous biological and mechanical complications, such as soft tissue inflammation, screw loosening, component fracture, and ceramic chipping. 10 Such complications have major maintenance and financial burdens on the patients and the treating clinicians. Therefore, it is essential to disclose all the variables that can influence the accuracy of digital implant impressions for multiunit implant prostheses.

The literature indicates that the implant digital impression is influenced by the scan body, 11 12 the number of implants, 2 13 14 the span of scanning, 2 3 15 16 17 the alignment, location and depth of implants, 5 6 14 18 the scanned surface morphology, and access to the relevant surfaces, 19 20 21 22 23 24 and the scanning path. 25 However, to the knowledge of authors, studies investigating the effect of adjacent teeth on digital scanning of implants are lacking in the literature. The presence and the condition of adjacent teeth to the implant scan body may influence the access to the scan body surface, merging of sequential IOS images, shadowing on the scan body, and manipulation and handling of the IOS camera. Therefore, this study aimed to evaluate the effect of adjacent teeth patterns on the accuracy of digital impressions of parallel and divergent implants placed for three-unit prostheses. The null hypotheses are the pattern of adjacent teeth that has no influence on the accuracy of digital impressions, and the digital impression for parallel and divergent implants exhibit similar accuracy.

## Materials and Methods

### Master Model Preparation

A maxillary typodont model (Nissin Dental Products Inc., Kyoto, Japan) was modified by removing the first premolars and first molars, and replacing them with tissue level implants (Straumann, Institut Straumann AG, Basel, Switzerland). The aim was to develop scenarios for two implants scanning. All implants had a regular diameter with a 4.8 mm neck diameter, and were fixed in the socket by embedding them in self-curing acrylic resin (GC Pattern Resin, GC Corp, Tokyo, Japan). On the left side, the implants were completely parallel, while the right-side implants had a 15-degree buccolingual divergence angle between them. The angle was achieved by tilting the first molar implant buccally. The parallelism and the divergence were confirmed by a dental surveyor.

**Fig. 1:**
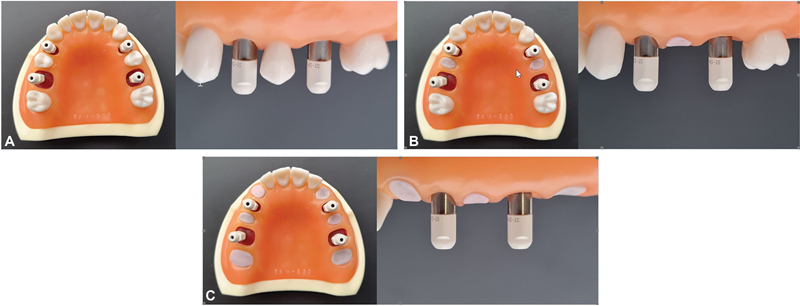
The simulated clinical scenarios with the parallel implants on the left side, and divergent implants on the right side. The divergence was achieved by tilting the right first molar implant 15 degrees buccally. ( **A** ) S1: Partially edentulous arch with missing first premolars and first molars. ( **B** ) S2: Partially edentulous arch with missing first premolars, second premolars, and first molars. ( **C** ) S3: Partially edentulous arch with missing canines, first premolars, second premolars, first molars, and second molars.

The modified maxillary model with the four implants was used as a master model that was further altered by removing adjacent teeth to the implants to simulate three clinical scenarios for two implants scanning ( Fig. 1 ): (1) Partially edentulous arch with missing first premolars and first molars (S1), (2) partially edentulous arch with missing first premolars, second premolars and first molars (S2), and (3) partially edentulous arch with missing canines, first premolars, second premolars, first molars, and second molars (S3). After removing the adjacent teeth, the socket area was sealed with laboratory putty (Coltene Whaledent, Altstatten, Switzerland).

### Digital Impression

IOS (Trios 4, 3Shape, Copenhagen, Denmark) was used to make the digital scans. To simulate the clinical environment, the digital impressions were performed in a phantom head with an opposing dentate mandibular model. Intraoral scan bodies (ZFX Scan body, ZFX Dental, Zimmer Biomet, Warsaw, Indiana, United States) compatible with Straumann tissue level implants were connected to the implants. Prior to digital impression, the scanner was calibrated, and the manufacturers' instructions were followed. The scanning path was a zigzag motion to record the occlusal–palatal aspects followed by the buccal surfaces. A total of 10 scans ( *n* = 10) were obtained for each scenario (S1, S2, and S3) and for each side (parallel and divergent implants). All scanned models were exported into STL format. The scanning span for all the groups was similar and captured the complete quadrant.

### Accuracy Evaluation

**Fig. 2:**
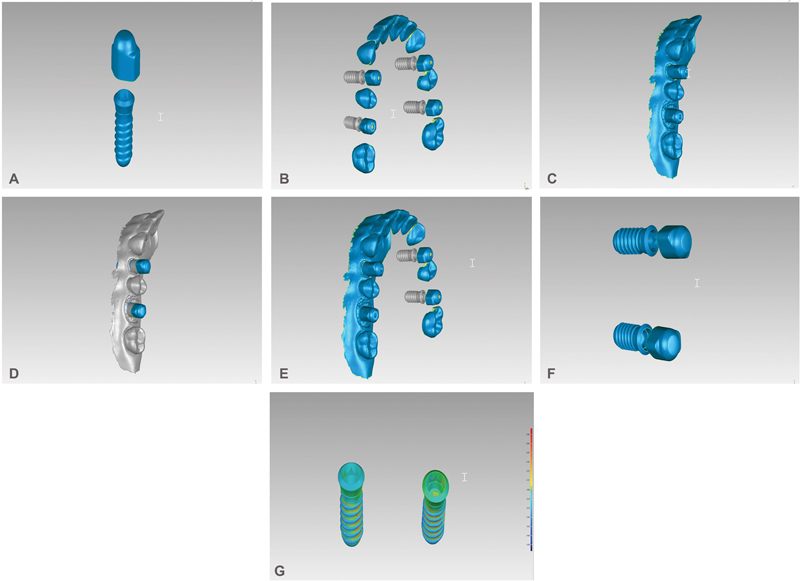
( **A** ) The reverse engineered virtual scan body with a virtual implant template that was used to determine the implant position. ( **B** ) The master reference model with the determined virtual implants. ( **C** ) An example of the scanned model. ( **D** ) The virtual position of the implants was determined by superimposition the virtual scan body and implant template on the scanned model. ( **E** ) The modified scanned model was superimposed on the master model. ( **F** ) The virtual implants were extracted and the unnecessary tissue were removed. ( **G** ) Subsequently, the extracted implants of the scanned model and virtual model we analyzed.

To generate a virtual reference model, the master model with attached scan bodies was scanned by a laboratory scanner (Identica T300, Medit Identica, DT Technologies, Davenport, Iowa, United States). The virtual reference model was used to evaluate the accuracy of each scanned model. A virtual scan body with a virtual implant template was reverse engineered ( Fig. 2A ), and was used as a key to convert the reference model and each scanned model to virtual implants. A three-dimensional (3D) rendering software (Geomagic Control, 3D systems, Rock Hill, South Carolina, United States) was used to manipulate the virtual models. Through the superimposition process, for each implant, a scan body and virtual implant template was superimposed on the scan body surface of the scanned model ( Fig. 2D ). This was achieved by selecting three well-distributed points on the scan body surface followed by automated iteration for best fit alignment. Subsequently, all the surfaces were deleted except the virtual implant bodies ( Fig. 2F ). Therefore, each image was converted to 2 virtual implants without the surrounding structure ( Fig. 2G ).

Three accuracy variables were measured for every group: trueness, precision, and interimplant distance. All the measurements were calculated via the Geomagic Control software. The trueness and precision were superimposition-related variables and followed ISO 5725 standards, where trueness is the deviation of the implants of the test images from the reference image ( *n* = 10), and precision is the deviation between the implants of the different test images within the same group ( *n* = 45). The absolute deviation of randomly distributed points on the implants surfaces was used to calculate the root mean square (RMS) value using the following equation:



where *Ri* is the spatial point of the reference image, *Ci* is the same spatial point of the test image, and *n* is the total number of points. The less the RMS, the greater the trueness and precision.

The interimplant distance was virtually measured between the centers of the implant platforms at each side of every virtual image. The interimplant distance deviation was the difference between the interimplant distances of the test and reference images.

### Statistics

The Shapiro–Wilk test was employed to confirm normality of the data. The one-way analysis of variance test followed by the Tukey Honest Significant Difference post-hoc test was used for each master model to evaluate the difference among the different clinical scenarios. In addition, for each group, the divergent implants were compared against the parallel implants using the *t* -test. All the tests were performed using a statistics program (SPSS for Windows, v23; SPSS Inc., Chicago, Illinois, United States), with a 0.05 level of significance.

## Results

### Trueness

**Fig. 3:**
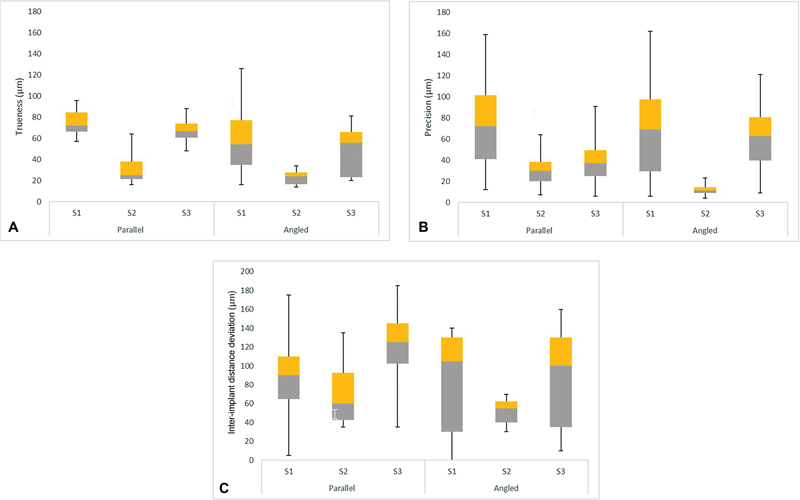
Box and Whisker plots summarizing the accuracy (μm) of parallel and divergent implants for the different scenarios. ( **A** ) Trueness, ( **B** ) precision, and ( **C** ) interimplant distance.

For the parallel implants, the best trueness was observed for S2 (mean = 30.0 µm, standard deviation [SD] = 14.3 µm), followed by S3 (mean = 67.3 µm, SD = 11.3 µm) and S1 (mean = 74.8 µm, SD = 11.8 µm) ( *p* < 0.001) ( Fig. 3A ). Significant differences existed between S1 and S2 ( *p* < 0.001), and S2 and S3 ( *p* < 0.001). The S1 and S3 were generally similar ( *p* = 0.39). Likewise, significant differences existed within the divergent implants ( *p* = 0.007), where S2 (mean = 23.1 µm, SD = 7.1 µm) had the best trueness followed by S3 (mean = 48.2 µm, SD = 23.0 µm) and S1 (mean = 59.4 µm, SD = 34.0 µm). Significant differences existed only between S1 and S2 ( *p* = 0.006), and no significant difference was detected between S1 and S3 ( *p* = 0.56) and S2 and S3 ( *p* = 0.07).

While parallel implants appeared to have inferior trueness than divergent implants, no significant difference was observed for any of the comparisons (S1 = 0.48, S2 = 0.97, S3 = 0.24).

### Precision

The precision showed a generally similar pattern to trueness. S2 had the most superior precision (mean = 31.3 µm, SD = 15.7 µm) followed by S3 (mean = 38.0 µm, SD = 18.0 µm) and S1 (mean = 70.3 µm, SD = 37.3 µm) for parallel implants ( *p* < 0.001) ( Fig. 3B ). Significant differences existed between S1 and S2 ( *p* < 0.001), and S2 and S3 ( *p* < 0.001). The S1 and S3 were generally similar ( *p* = 0.43). The divergent implants exhibited a similar precision pattern to parallel implants, where significant differences existed between the different groups ( *p* < 0.001). Specifically, the S2 had the best precision (mean = 12.3 µm, SD = 4.7 µm) followed by S3 (mean = 62.1 µm, SD = 26.5 µm) and S1 (mean = 66.9 µm, SD = 39.2 µm). Significant differences existed between S1 and S2 ( *p* < 0.001), and S2 and S3 ( *p* < 0.001). No significant difference was detected between S1 and S3 ( *p* = 0.69).

The parallel implants were similar in precision to divergent implants for S1 ( *p* = 0.99). However, the parallel implants were significantly inferior to divergent implant for S2 ( *p* = 0.01), and divergent implants were inferior to parallel implants for S3 ( *p* < 0.001).

### Interimplant Distance Deviation

For parallel implants, the S2 had the least interimplant distance deviation (mean = 70.0 µm, SD = 33.4 µm), followed by S1 (mean = 88.0 µm, SD = 46.7 µm) and S3 (mean = 121.0 µm, SD = 39.5 µm) ( *p* = 0.03) ( Fig. 3C ). The difference was significant between S1 and S3 ( *p* = 0.01), and S2 and S3 ( *p* = 0.02). The S1 and S2 were similar ( *p* = 0.22). Likewise, for divergent implants, the S2 was most superior (mean = 53.0 µm, SD = 13.4 µm), while the S1 (mean = 88.0 µm, SD = 49.4 µm) and S3 (mean = 85.0 µm, SD = 53.2 µm) were similar. However, the differences among the three scenarios were not significant for the divergent implants ( *p* = 0.15).

The difference between the parallel and divergent implants was not significant for S1 ( *p* = 0.5), S2 ( *p* = 0.05) and S3 ( *p* = 0.08).

## Discussion

The aim of the study was to evaluate the effect of adjacent teeth patterns on the accuracy of digital impressions of parallel and divergent implants placed for three-unit prostheses. The hypothesis that the pattern of adjacent teeth has no influence on the accuracy of digital impressions was rejected, and the hypothesis that the digital impressions for parallel and divergent implants exhibit similar accuracy was accepted. In general, the most superior outcome for all the variables was observed for S2. This indicates that for two implants scanning, adjacent teeth to the scanning span enhance the accuracy of digital implant impression, as long as the interimplant area is clear. In addition, the presence of an angle between the implants appears to have a minimal effect on the accuracy, magnitude, and pattern of errors. For each group, the parallel and the divergent implants showed a similar pattern and magnitude of deviation. This observation confirms that implant angulation does not influence the accuracy of digital impression. 1 4 5 6 9

The superior outcome of S2 over S1 and S3 can be attributed to the pattern of the remaining adjacent teeth and their influence on the visibility of implant scan bodies during digital impression. For example, with the missing tooth between the two implants, the scan body surfaces were more accessible for scanning at the interimplant area and the IOS camera light is likely to be projected perpendicularly, which improves the accuracy of surface scanning. 20 21 22 23 24 As reported in previous studies, 25 26 27 the presence of a tooth or solid marker serving as a reference for automated IOS stitching did not improve the accuracy of digital impressions. In the present study, this can be related to the reduced visibility of the scan body for the S1 situation. Specifically, the presence of a tooth between the two implants resulted in the axial surfaces of the tooth and the scan bodies facing each other at a close distance that caused shadowing on the scan bodies. As a result, perpendicular scanning of the scan body surfaces was only possible on the occlusal, buccal, and lingual surfaces. Proximal surfaces were scanned at an angle that mandated more software estimation of the missing data resulting in loss of surface details. 2 3 This is even more complicated if the scan body has undercuts 3 as the ones used in this study. Scanning at an angle was associated with more surface deviations and errors that will eventually lead to reduced registration accuracy of the implant positions. 1 19 Another contributing factor to the reduced accuracy for the S1 is that the teeth had a similar height to the scan bodies, which further obscured the proximal surfaces. To overcome this limitation, the visibility of the scan body can be improved by increasing the length of the scan body to project beyond the occlusal surface of the adjacent teeth. Earlier studies indicated that shallower implants scanned with completely visible scan bodies had a higher accuracy than deeper implants with partially visible scan bodies, 6 18 regardless of the angulation. 5 Therefore, it is reasonable to consider strategies to modify the digital impression procedure to enable greater visibility of the scan bodies during scanning and to reduce the proximal shadowing, such as the use of narrower and longer scan body design.

The presence of mesial and distal teeth, as for S1 and S2, appears to provide an advantage over S3. This could be related to the more defined features for scanning and subsequent registration, leading to more rigid image stitching and surface reconstruction. The S3 scenario is disadvantaged by the larger edentulous span, free end edentulous presentation, and less defined reference surfaces. 13 14 The presence of teeth next to the scan bodies served as prominent landmarks and were shown to improve the quality of stitching. 14 Several earlier studies revealed that the longer the spanning length, the greater the errors in trueness and precision. Specifically, the increased length of the edentulous area and scanning span decreased the accuracy. 2 3 15 16 Increasing the span of scanning will increase the amount of stitching images and the reliance on the software algorithm to refine the final image. 6 16 28 Nevertheless, most of the studies on the negative effect of scanning span were on whole arch scanning, and segmental scanning was shown to be acceptable and comparable to conventional implant impressions. 2 3 14 16

While the different scenarios can influence the accuracy of digital impressions, the errors of all the techniques are within the claimed clinically acceptable level range of 30 to 150 µm. 1 2 29 The observed errors ranged from 50 to 120 µm may neither be clinically detectible nor significant from the biological or mechanical perspective. 2 10 29 It is important to emphasize that the results of this study are only applicable to digital impression for short span implant prostheses on two implants. Although the presence of a tooth between two implants appears to challenge the digital impression, clinically they will be restored by separate single crowns, and relating the position of the implant to the neighboring implant for the S1 situation is less relevant than for S2 and S3 presentations, where the prosthesis will splint the implants. The general recommendation is in line with the earlier laboratory studies that whenever the visibility of the scan body is affected, it is worthy to consider adjunctive procedures to enhance the accuracy of digital impression. 26 30 31 32 33 This involves modifying the scanning pattern, 25 scan body design and length, 30 or placements of additional landmarks. 26

Despite the attempts of this study to simulate clinical scenarios by scanning in a phantom head, several clinical variables were impossible to simulate. For example, the edentulous ridges were composed of a silicone surface that was neither wet nor smooth in comparison to natural oral mucosa. In addition, the sockets of the removed teeth were sealed with silicone plugs that may further facilitate the scanning process. The lack of natural buccal tissue, saliva, and patient movement would have simplified the scanning process. In addition, the outcome of the present study is only relevant to the tested IOS system and scan bodies. Different IOS system scan bodies may reveal a different result. 12

## Conclusion

Within the limitations of this study, it was concluded that digital implant scans for three-unit prostheses appeared to be clinically accurate regardless of the clinical scenario. Clear interabutment area improved the accuracy of the digital scans. In addition, buccolingual angle between implants had no influence on the accuracy of the digital scans.
